# Left atrial thrombus following bilobectomy: a case report

**DOI:** 10.1186/1752-1947-4-71

**Published:** 2010-02-24

**Authors:** Onder Teskin, Yeşim Bicer, Ugur Kaya, Sertac Cicek

**Affiliations:** 1 Division of Cardiovascular Surgery and Anesthesiology, Acibadem Hospital, Bursa, Turkey

## Abstract

**Introduction:**

Left atrial free floating ball thrombus is a relatively rare event, especially without mitral valve disease.

**Case presentation:**

A 61-year-old Turkish man was admitted to our hospital with a thrombus mass in his left atrium. Five months earlier, he had undergone right bilobectomy and superior bronchoplasty due to squamous cell carcinoma in the lung. The patient had no evidence of cardiac disease except atrial fibrillation and there were no defined embolizations. The thrombus mass was surgically removed. The patient was discharged from hospital on the sixth postoperative day.

**Conclusion:**

Surgery with cardiopulmonary bypass is a safe method for treatment. The patient should be medicated with warfarin, especially in the presence of atrial fibrillation.

## Introduction

A free thrombus in the left atrium without concomitant mitral valve disease is a rare finding. This report presents a patient who developed progressive dyspnea five months after right bilobectomy. A left atrial thrombus was diagnosed after computed tomography (CT) and transesophageal echocardiography.

The first case with left atrial thrombus was reported in 1814. Currently, the use of CT, magnetic resonance imaging (MRI) and echocardiography have made the diagnosis much easier.

## Case presentation

**Figure 1:**
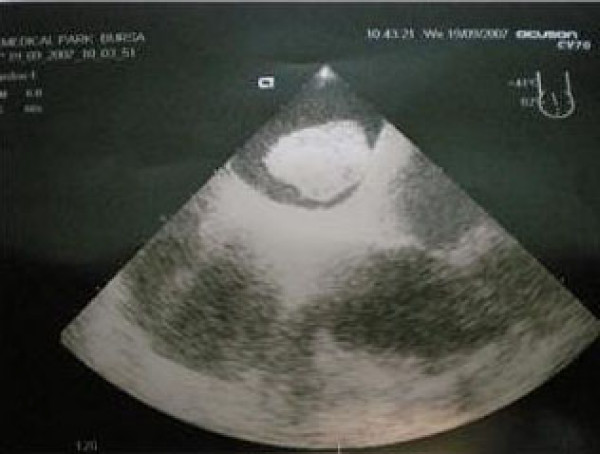
**Left atrial thrombus in mid-esophageal sections**.

The patient, a 61-year-old Turkish man, had undergone right bilobectomy and superior bronchoplasty due to squamous cell carcinoma in the lung five months earlier. He received seven sessions of chemotherapy in the postoperative period. In the last month, he started to experience dyspnea which increased progressively. During control measurements it was seen from electrocardiogram (ECG) findings that he had atrial fibrillation and left bundle branch block. His control thorax CT showed a mass in the left atrium. Further diagnosis was performed with transesophageal echocardiography and the image was diagnosed as a 50 × 60 mm thrombus (Figure 1). There was no concomitant mitral valve pathology.

**Figure 2:**
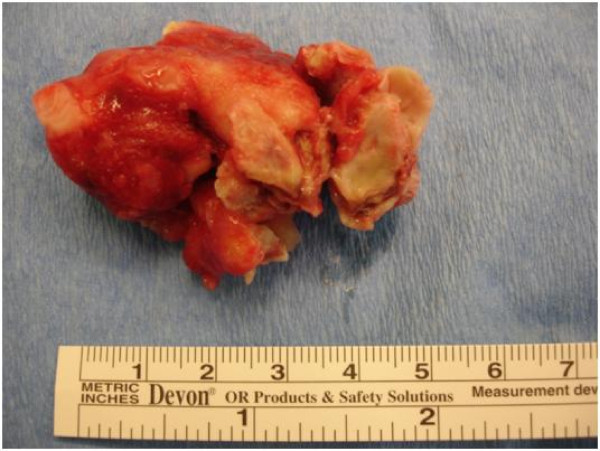
**Mass extirpated from the left atrium**.

The surgery was performed by our team under cardiopulmonary bypass with mild hypothermia (34°C); left atriotomy was carried out and the 60 × 40 × 40 mm thrombus mass, located in the left atrium and partly in the right upper pulmonary vein, was extirpated (Figure 2). No complication was encountered during and after the operation. Pathologic examination showed an organized thrombus. He was discharged from the hospital on the 6th postoperative day and was medicated with warfarin, acetylsalicylic acid and digitalis. A form about ethnicity was signed by patient.

## Discussion

It is a rare finding to see a left atrial thrombus without concomitant mitral valve disease. A left atrial ball thrombus in a non-rheumatic patient with atrial fibrillation was first described in 1992 [1] and in the first published reports, the symptoms were due to thromboembolism.

Transesophageal echocardiography is a very sensitive diagnostic method for diagnosis of left atrial thrombus. Atrial fibrillation is almost always an accompanying finding, and mitral stenosis, a history of previous mitral valve procedures, myocardial infarction, hypertrophic cardiomyopathy, or infective endocarditis may be other accompanying conditions [2].

The etiology in cases without additional cardiac disorders or atrial fibrillation is not clear. In our patient, thrombosis may have been triggered by the surgical trauma of right bilobectomy superior bronchoplasty. Pulmonary vein thrombosis after pulmonary vein resection is also a rare complication [3]. The pathophysiology may be growth of the thrombus in the left atrium and taking on the shape of the cavity, and then becoming a pedunculated mobile mass [2]. The free thrombus in the left atrium can be highly thromboembolic [4].

Metastatic tumors should be the pathology of differential diagnosis [5]. Tumoral embolizations due to pulmonary resection may occur in the cerebral circulation, mitral valve, left ventricular outflow tract, coronaries, aortic bifurcation and the extremities [6]. Tumor fragments may localize in the pulmonary vein stump, left atrium, left ventricular trabeculae or chorda tendinea after pulmonary resection and may cause late embolization [7].

In our patient, there were no defined embolizations. As there is a high risk of embolization, the symptoms of emboli (such as mesenteric ischemia; abdominal pain) should alert the surgeon that urgent treatment is required. It is worth remembering that most of the embolizations occur during or after pulmonary resection [5].

Left atrial and pulmonary vein thrombi are a high risk for thromboemboli with a high mortality rate [2]. Surgical extirpation of the thrombus is strongly advised. Anticoagulation and thrombolytic therapies do not appear to have a role in the acute management of left atrial ball thrombus [8]. Surgery with cardiopulmonary bypass is a safe method for treatment. The patient should be medicated with warfarin, especially in the presence of atrial fibrillation.

## Abbreviations

CT: computed tomography; MRI: magnetic resonance imaging; ECG: electrocardiogram.

## Consent

Written informed consent was obtained from the patient for publication of this case report and any accompanying images. A copy of the written consent is available for review by the Editor-in-Chief of this journal.

## Competing interests

The authors declare that they have no competing interests.

## Authors' contributions

TO was a major contributor in collecting data, writing and preparing the manuscript. KU was involved in surgical team. BY was involved in operation team as anesthetist. CS performed the surgical excision and was involved in editing the manuscript. All authors read and approved the final manuscript.
